# Holiday gatherings, mobility and SARS-CoV-2 transmission: results from 10 US states following Thanksgiving

**DOI:** 10.1038/s41598-021-96779-6

**Published:** 2021-08-30

**Authors:** Shruti H. Mehta, Steven J. Clipman, Amy Wesolowski, Sunil S. Solomon

**Affiliations:** 1 Department of Epidemiology, Johns Hopkins Bloomberg School of Public Health, Baltimore, MD USA; 2 Department of International Health, Johns Hopkins Bloomberg School of Public Health, Baltimore, MD USA; 3 Division of Infectious Diseases, Department of Medicine, Johns Hopkins University School of Medicine, Baltimore, MD USA; 4 Department of Epidemiology, Johns Hopkins Bloomberg School of Public Health, 615 N. Wolfe Street, Room E6546, Baltimore, MD 21205 USA

**Keywords:** Epidemiology, Infectious diseases

## Abstract

Public health officials discouraged travel and non-household gatherings for Thanksgiving, but data suggests that travel increased over the holidays. The objective of this analysis was to assess associations between holiday gatherings and SARS-CoV-2 positivity in the weeks following Thanksgiving. Using an online survey, we sampled 7770 individuals across 10 US states from December 4–18, 2020, about 8–22 days post-Thanksgiving. Participants were asked about Thanksgiving, COVID-19 symptoms, and SARS-CoV-2 testing and positivity in the prior 2 weeks. Logistic regression was used to identify factors associated with SARS-CoV-2 positivity and COVID-19 symptoms in the weeks following Thanksgiving. An activity score measured the total number of non-essential activities an individual participated in the prior 2 weeks. The probability of community transmission was estimated using Markov Chain Monte Carlo (MCMC) methods. While 47.2% had Thanksgiving at home with household members, 26.9% had guests and 25.9% traveled. There was a statistically significant interaction between how people spent Thanksgiving, the frequency of activities, and SARS-CoV-2 test positivity in the prior 2 weeks (p < 0.05). Those who had guests for Thanksgiving or traveled were only more likely to test positive for SARS-CoV-2 if they also had high activity (e.g., participated in > one non-essential activity/day in the prior 2 weeks). Had individuals limited the number and frequency of activities post-Thanksgiving, cases in surveyed individuals would be reduced by > 50%. As travel continues to increase and the more contagious Delta variant starts to dominate transmission, it is critical to promote how to gather in a “low-risk” manner (e.g., minimize other non-essential activities) to mitigate the need for nationwide shelter-at-home orders.

## Introduction

As the COVID-19 pandemic surged across the United States in November 2020, public health officials strongly cautioned, and indeed recommended, against travel and social gatherings for Thanksgiving^1^. Data from TSA, however, suggested that travel during the week of Thanksgiving was the highest since the beginning of the pandemic, representing only a 40% reduction from 2019 Thanksgiving travel^2^. Vehicle travel also peaked during the holiday week^3,4^. While not all US states experienced post-Thanksgiving surges in case counts, hospitalizations and deaths equally, the US as a whole which was already on an increasing trajectory experienced its then highest daily new infection rate (220,290 new infections on a single day) in mid-December just 3 weeks post the Thanksgiving holiday^5^. With this escalation of cases, hospitalizations, and deaths, concerns increased in anticipation of December holiday travel. The Centers for Disease Control and Prevention (CDC) issued similar guidance for the December holidays recommending staying home and/or postponing travel and offering guidance for precautions to be taken in the setting of planned travel^6^. Data from TSA again showed increased travel during late December with an average of over 1 million travelers per day from December 18, 2020–January 4, 2021^2^. Subsequently, the US experienced another surge in infections with a peak of 249,836 cases in a single day in the second week of January^5^.

While the observed infection surges appear to be related to increased travel and gatherings around the holidays, it is difficult to draw conclusions based purely on ecological data. Indeed, the US was already on an increasing trajectory of infections prior to the Thanksgiving holiday^5^. There has been little data collected on individual-level behaviors around the holidays which could not only help us to better understand the impact of holiday travel and gatherings but also might provide guidance to public health officials about how to manage future messaging particularly as travel undoubtedly increases globally and we encounter third and fourth waves of the pandemic. The aim of this analysis was to examine the association of Thanksgiving gatherings and travel with SARS-CoV-2 positivity and COVID-19 symptoms in a sample of nearly 8000 persons across 10 diverse US states. We further evaluated interactions between Thanksgiving gatherings/travel and participation in other activities and modeled the impact of travel and gatherings on community SARS-CoV-2 transmission in order to examine how much transmission might have been prevented by limiting travel/gatherings.

## Results

### Demographics

The median age of the 7770 persons in the sample was 46 years (interquartile range [IQR]: 32–62); about half (49.3%) were female (Table 1). The majority (55.1%) were White, 12.8% were Black/African American; 25.0% self-reported Hispanic/Latino ethnicity. The median household size was 2 (IQR, 2–4). A total of 2990 (38.2%) reported working outside the home, of whom 1302 were considered essential/front-line workers.

**Table 1:** Description of study population by Thanksgiving travel, 7700 adults sampled across 10 US states from December 4–18, 2020.

.	Thanksgiving at home with household members only	Thanksgiving at home with ≥ 1 non-household member	Traveled outside the home for Thanksgiving	p value	Overall
	n = 3652	n = 2063	n = 2055		n = 7770
Median age in years, (IQR)	49 (35, 65)	43 (29, 60)	41 (30, 57)	<0.001	46 (32, 62)
Male gender	1824 (51.0)	1035 (50.3)	1047 (50.5)	0.76	3906 (50.7)
**Race/ethnicity**	−	−	−	<0.001	−
White	1948 (54.8)	1032 (51.8)	1182 (59.1)		4162 (55.1)
Black/African American	506 (13.6)	293 (13.9)	215 (10.4)		1014 (12.8)
Hispanic/Latino	781 (22.6)	582 (28.2)	542 (26.0)		1905 (25.0)
Asian/Pacific Islander	326 (8.71)	110 (5.84)	82 (4.34)		518 (6.80)
Other	69 (0.32)	35 (0.26)	29 (0.15)		133 (0.36)
**Annual household income**	−	−	−	0.06	−
< $20,000	504 (16.9)	277 (15.4)	229 (13.2)		1010 (15.5)
$20,000–39,000	614 (18.4)	348 (17.5)	354 (17.8)		1316 (18.0)
$40,000–49,000	308 (7.67)	169 (7.51)	196 (9.46)		673 (8.10)
$50,000—$69,000	622 (17.3)	335 (16.6)	373 (17.2)		1330 (17.1)
≥ $70,000	1355 (39.9)	819 (43.0)	815 (42.3)		2989 (41.4)
Median household size (IQR)	2 (2, 4)	2 (2, 4)	2 (2, 4)	0.21	2 (2, 4)
Children ≤ 18 years of age in the home	1061 (30.0)	728 (35.7)	804 (40.8)	<0.001	2593 (34.4)
Persons ≥ 65 years of age in the home	968 (26.6)	462 (22.1)	331 (17.2)	<0.001	1761 (22.9)
**Employment**	−	−	−	<0.001	−
Working at home	1174 (32.7)	615 (30.2)	535 (25.9)		2324 (30.3)
Not working	1319 (36.4)	594 (30.1)	537 (27.8)		2450 (32.5)
Working outside home (non-essential)	700 (20.1)	465 (23.2)	501 (24.7)		1666 (22.1)
Essential (healthcare)	164 (4.15)	150 (6.71)	203 (9.17)		517 (6.14)
Essential (other)	266 (6.73)	221 (9.74)	273 (12.4)		760 (9.01)
**State of residence**				< 0.001	
Massachusetts	400 (10.7)	216 (10.2)	220 (11.6)		836 (10.8)
Maryland	377 (10.0)	195 (10.1)	168 (7.99)		740 (9.52)
Illinois	531 (14.5)	267 (13.1)	246 (11.9)		1044 (13.4)
Wisconsin	353 (9.59)	188 (9.37)	202 (9.76)		743 (9.58)
Florida	418 (11.7)	261 (12.6)	277 (13.0)		956 (12.3)
Texas	617 (17.1)	425 (20.3)	425 (20.6)		1467 (18.9)
California	727 (19.9)	364 (17.5)	363 (17.9)		1454 (18.7)
Nebraska	125 (3.68)	93 (4.02)	82 (3.92)		300 (3.84)
North and South Dakota	104 (2.83)	54 (2.79)	72 (3.40)		230 (2.97)

Data are presented as n (%) unless otherwise specified. Numbers may not sum to total if there were participants who elected not to answer a given question. P values from Kruskal–Wallis tests for continuous variables and chi-squared tests for categorical variables using unweighted estimates.

IQR, interquartile range.

### Thanksgiving and SARS-CoV-2 positivity

Overall, 2055 (26.5%) of 7770 reported having Thanksgiving outside of their home with minimal variability by state (21.7% in Maryland to 29.6% in the Dakotas). Of those who had Thanksgiving outside their home, most (90.5%) reported traveling by car with 3.7% traveling by plane. An additional 2063 (26.6%) had Thanksgiving at home but with at least one person from outside their household. The median Thanksgiving dinner size for those who had Thanksgiving outside of their home was 6 (IQR: 3–9) compared to 4 (IQR: 3–7) for those who had Thanksgiving at home with ≥ 1 non-household member and 2 (IQR: 1–3) for those who had Thanksgiving with their household only (p < 0.001).

Compared to those who had Thanksgiving with their household, those who had Thanksgiving with others or outside their home were significantly more likely to report COVID-19 symptoms, testing for SARS-CoV-2, and testing positive for SARS-CoV-2 infection all in the prior 2 weeks, with the highest levels in those who had Thanksgiving away from home (Table 2**,** Supplementary Figure 1). Among those tested in the prior 2 weeks, those who had Thanksgiving outside their home were significantly more likely to self-report a positive SARS-CoV-2 result compared to those who had Thanksgiving at home with or without non-household members (41.2% vs. 25.5% and 13.3%, respectively; p < 0.001).

**Table 2:** Thanksgiving travel, activity and SARS-CoV-2 testing history among 7770 adults sampled across 10 US states from December 4–18, 2020.

	Thanksgiving at home with household members only	Thanksgiving at home with ≥ 1 non-household member	Traveled outside the home for Thanksgiving	p value	Overall
	n = 3652	n = 2063	n = 2055		n = 7770
**Mode of Thanksgiving travel**	−	−	−	<0.001	−
Car	−	−	1750 (90.2)		−
RV	−	−	28 (1.66)		−
Train/bus	−	−	72 (4.12)		−
Plane	−	−	72 (3.39)		−
Boat	−	−	11 (0.66)		−
Median size of Thanksgiving dinner (IQR)	2 (1, 3)	4 (3, 7)	6 (3, 9)	<0.001	3 (1,6)
COVID-19 symptoms^a^	235 (6.40)	178 (9.34)	344 (16.1)	<0.001	757 (9.69)
Tested for SARS-CoV-2^a^	313 (8.59)	227 (10.9)	470 (21.8)	<0.001	1010 (12.6)
Tested positive for SARS-CoV-2^a,b^	41 (13.3)	47 (25.5)	185 (41.2)	<0.001	273 (28.6)
**SARS-CoV-2 positivity in household members**^**a**^	−	−	−	<0.001	−
None	3225 (97.5)	1790 (93.0)	1582 (87.5)		6,597 (93.7)
One	51 (1.47)	71 (3.73)	164 (7.69)		286 (3.67)
More than one	31 (1.06)	61 (3.31)	93 (4.83)		185 (2.64)
Median visits to grocery store/pharmacy (IQR)^a^	2 (1, 4)	2 (1, 4)	3 (2, 4)	<0.001	2 (1, 4)
Median number of non-essential activities in prior 2 weeks, (IQR)^a,c^	1 (0, 2)	2 (1, 6)	4 (2, 10)	<0.001	2 (0, 5)
Median hours spent visiting with friends and family (IQR)^a^	0 (0, 2)	3 (0, 10)	6 (2, 18)	<0.001	0 (0, 8)
**Masking when visiting with friends and family**^**a,d**^	−	−	−	<0.001	−
Never	289 (28.6)	410 (35.9)	595 (39.2)		1294 (35.2)
Sometimes	536 (51.1)	636 (51.3)	827 (50.1)		1999 (50.8)
Always	215 (20.3)	163 (12.8)	176 (10.8)		554 (14.1)

Numbers may not sum to total if there were participants who elected not to answer a given question. P values from Kruskal–Wallis tests for continuous variables and chi-squared tests for categorical variables using unweighted estimates.

IQR, interquartile range.

^a^Reflect behaviors in the prior 2 weeks.

^b^Among persons who received a SARS-CoV-2 PCR test in the prior 2 weeks.

^c^Non-essential activities included bars, restaurants, visits with friends/families, theatres, stadiums, fitness activities, gym visits and church.

^d^Among 3889 persons who reported visiting with friends or family either outdoors or indoors in the prior 2 weeks.

### Mobility/activity following Thanksgiving and SARS-CoV-2 positivity

Overall, persons went to a grocery store/pharmacy a median 2 times (IQR, 1–4) in the prior 2 weeks with limited variability by how they spent Thanksgiving (Table 2). By contrast, there was substantial variability in the number of non-essential activities a person participated in by how they spent Thanksgiving with those that had Thanksgiving away from home participating in a median of 4 non-essential activities (IQR, 2–10) in the prior 2 weeks compared to a median of 1 activity (IQR, 0–2) in those who had Thanksgiving with their household. Moreover, there was even more variability when considering both Thanksgiving and SARS-CoV-2 test positivity in the prior 2 weeks. Persons who had Thanksgiving outside their home and tested positive for SARS-CoV-2 participated in a median of 33 non-essential activities in the prior 2 weeks (IQR: 21–50; Fig. 1). By comparison, those who had Thanksgiving outside their home, but did not test positive participated in a median of 6 activities (IQR: 2–18) and those who had Thanksgiving with their household and tested negative for SARS-CoV-2 in the prior 2 weeks participated in a median 1 activity (IQR: 0–4).

**Figure 1:**
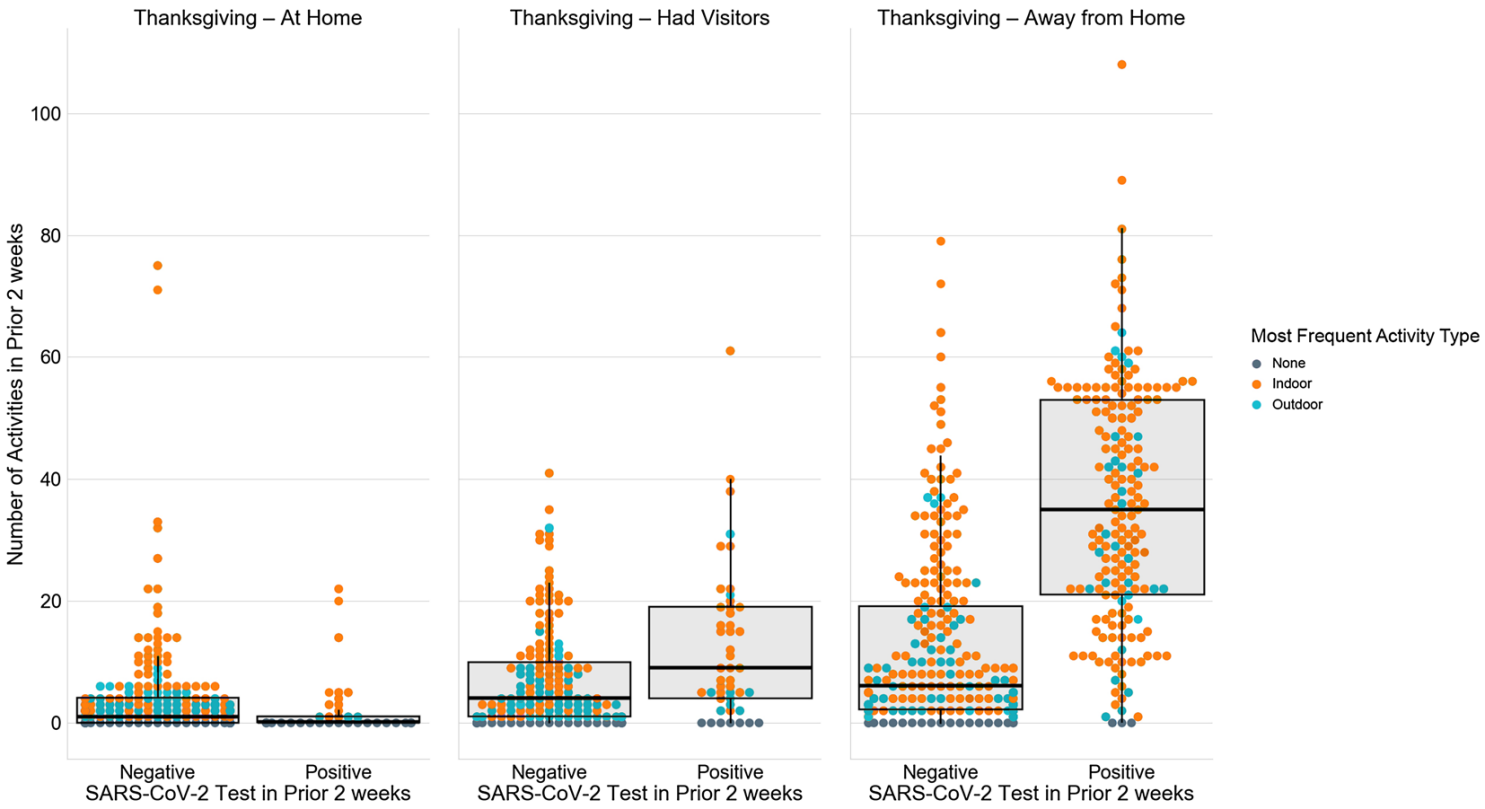
Summary of non-essential activities in the prior 2 weeks by Thanksgiving travel and SARS-CoV-2 test positivity in the prior 2 weeks.

In analyses that adjusted for age, gender, race/ethnicity, household size, employment, state of residence, and participating in non-Thanksgiving gatherings of 10 or more people in the prior 2 weeks, there was a statistically significant interaction between how people spent Thanksgiving and the frequency of other activities (p for interaction < 0.05, Table 3) and SARS-CoV-2 test positivity in the prior 2 weeks. Specifically, compared to those who had Thanksgiving with their household and had low activity defined as less than one non-essential activity per day in the prior 2 weeks, those with low activity scores who spent Thanksgiving with non-household members and those who spent Thanksgiving outside their home were no more likely to self-report positivity for SARS-CoV2- infection. However, those with high activity defined as one or more non-essential activities per day were significantly more likely to report positivity for SARS-CoV-2 if they had Thanksgiving with non-household members (adjusted odds ratio [AOR], 2.94; 95% confidence interval [CI] 1.43, 6.08) or had Thanksgiving outside their home (AOR, 5.99; 95% CI 3.41, 10.5). A similar statistically significant interaction was observed for self-reported COVID-19 symptoms (Supplementary Table 2).

**Table 3:** Factors associated with self-reported SARS-CoV-2 test positivity and self-reported symptoms in the prior 2 weeks.

	AOR for SARS-CoV-2 positivity (95% CI)^a^	AOR for COVID-19 symptoms (95% CI)
Age (per 5 years)	0.99 (0.93, 1.07)	0.89 (0.87, 0.92)
Male gender	0.94 (0.65, 1.35)	1.06 (0.89, 1.26)
**Race/ethnicity**		
White	1	1
Black/African American	0.90 (0.51, 1.61)	
Hispanic/Latino	1.16 (0.76, 1.76)	1.19 (0.95, 1.48)
Asian/Pacific Islander	1.57 (0.68, 3.63)	0.69 (0.45, 1.06)
Other	1.79 (0.41, 3.50)	1.51 (0.83, 2.71)
Household size (per person)	1.06 (0.96, 1.18)	1.02 (0.98, 1.06)
**Employment**		
Working at home	1	1
Not working	1.06 (0.55, 2.02)	1.09 (0.85, 1.39)
Working outside home (non-essential)	1.19 (0.70, 2.03)	0.94 (0.73, 1.20)
Essential (healthcare)	1.79 (1.04, 3.10)	2.23 (1.67, 2.97)
Essential (other)	2.06 (1.21, 3.50)	1.67 (1.28, 2.17)
Attending a gathering of 10 or more persons^b^	1.21 (1.09, 1.33)	1.16 (1.10, 1.23)
**Interaction between Thanksgiving and activity score**^**c**^		
Household members only, low activity	1	1
Non-household member, low activity	1.06 (0.61, 1.85)	0.97 (0.77, 1.22)
Outside home, low activity	0.84 (0.48, 1.47)	1.05 (0.82, 1.33)
Household members only, high activity	0.61 (0.13, 2.96)	1.76 (0.97, 3.19)
Non-household members, high activity	2.94 (1.43, 6.08)	2.22 (1.51, 3.28)
Outside home, high activity	5.99 (3.41, 10.5)	6.70 (4.98, 9.01)
**State of residence**		
Massachusetts	1	1
Maryland	1.31 (0.57, 3.01)	1.34 (0.86, 2.09)
Illinois	2.37 (1.07, 5.26)	1.87 (1.26, 2.78)
Wisconsin	1.79 (0.75, 4.28)	1.84 (1.21, 2.81)
Florida	1.58 (0.74, 3.37)	1.58 (1.06, 2.37)
Texas	2.44 (1.21, 4.89)	1.65 (1.13, 2.40)
California	2.24 (1.11, 4.50)	2.11 (1.45. 3.08)
Nebraska	3.46 (1.27, 9.40)	2.28 (1.38, 3.76)
North and South Dakota	0.40 (0.08, 2.16)	1.69 (0.94, 3.03)

AOR, adjusted odds ratio; CI, confidence interval.

^a^Among 962 tested in prior 2 weeks.

^b^Reflects behavior in the prior 2 weeks and excludes Thanksgiving dinner.

^c^Activity score was calculated as the sum of all non-essential activities in the prior 2 weeks. Low activity was considered a frequency less than once/day and high activity was a frequency of once a day or more.

### December holiday plans

Overall, 1514 (20.6%) were planning to either travel for the December holidays or host persons from outside their household. Those who had Thanksgiving outside their home reported planning to spend the December holidays with significantly more people (median: 4; IQR: 2–7) than those who had Thanksgiving at their home (median: 2; IQR: 1–4) (p < 0.001). Notably, planned travel over the December holidays was most common among those who tested positive for SARS-CoV-2 in the prior 2 weeks (61.4%) compared with 20.3% of those who tested negative in the prior 2 weeks and 9.7% among those who were not tested.

### Implications for community transmission

**Figure 2:**
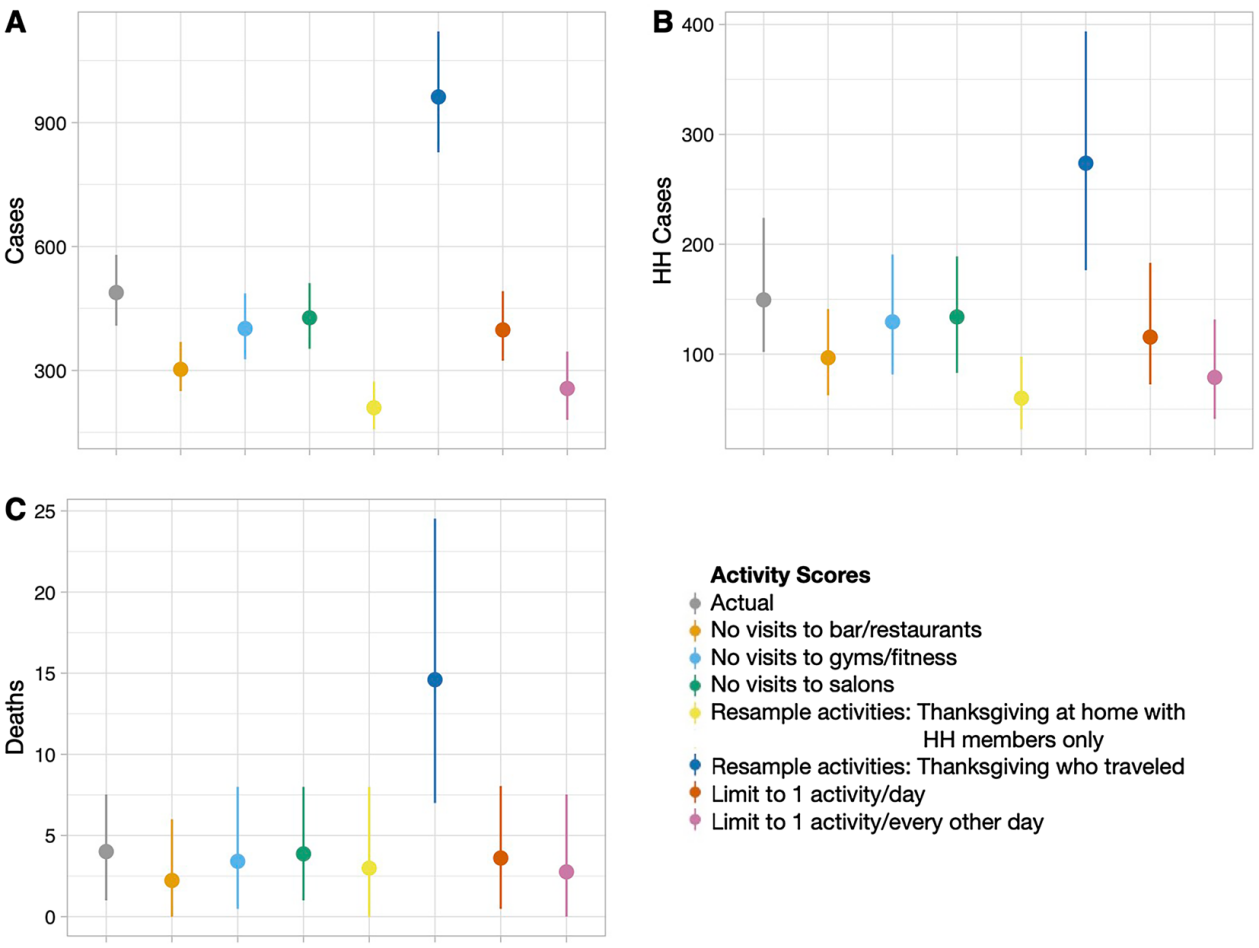
The modeled impact of activity patterns on SARS-CoV-2 positive cases. We estimated the (**A**) number of primary cases in surveyed individuals as well as (**B**) cases in their household contacts and (**C**) deaths in their household contacts. Mean values are shown as points with the 95% percentile range shown as lines Using the reported activity scores (grey) we also explored the impacts on a range of other activities including limiting activities, no visits to certain establishments, and different patterns of Thanksgiving celebrations.

Based on the reported activity patterns, we estimate that approximately 488 primary SARS-CoV-2 cases resulted from participants (95% percentile 408–579) in this sample and that this sample was responsible for 149 (95% percentile 102–224) secondary household cases and 4 deaths (95% percentile 1–7; Fig. 2). However, if all individuals in the sample had participated in only as many activities as those who had Thanksgiving at home with non-household members (the least active group), expected SARS-CoV-2 cases would have been reduced by more than 50% (mean = 210 cases, 95% percentile 157–273). By contrast, if all individuals in the sample had participated in the same number of activities as those who had Thanksgiving away from home (the most active group) we could have seen an increase of nearly two times the number of cases (mean = 962 cases, 95% percentile 828–1121). Policies that would have reduced the number of activities you could participate in (up to 7 or 14 activities) or by venue (no visits to gyms/outdoor fitness or bars/restaurants) would have a large impact on the number of cases with reductions on the order of 60–200+ primary cases. However, given the differences in the reported household structure, we did not observe a difference in the number of estimated deaths (Supplementary Data 1).

## Discussion

In this online survey that was conducted in the weeks following the 2020 Thanksgiving holiday, we found that the majority of persons sampled across 10 US states did not travel over the Thanksgiving holiday. However, we identified that those who did travel for Thanksgiving had a test positivity rate higher than that reported in any state in the US at the time of our survey and one that was significantly higher than those who did not travel. What was even more striking and somewhat surprising was that these individuals who traveled for Thanksgiving were significantly more active in the weeks immediately following Thanksgiving with frequent visits to bars, restaurants, and gyms among other places, and that the combination of Thanksgiving travel and participation in multiple activities post-Thanksgiving was associated with high SARS-CoV-2 test positivity and COVID-19 symptoms. While we cannot definitively say whether SARS-CoV-2 infection was a result of Thanksgiving travel or participation in multiple non-essential activities, it is clear that this small group of individuals may sustain ongoing SARS-CoV-2 transmission by potentially acting as “super spreaders”^7^.

While much was reported in the popular press about the surge of cases in the US post-Thanksgiving^5^ and other holidays, we are not aware of any empirical reports specifically describing behaviors around the holidays and associations with SARS-CoV-2 case positivity. Our data add to the primarily ecologic data on case counts^5^ with a diverse group of states at differing epidemic stages and with some variability in policies with respect to social distancing and masking—these states also, currently, also represent states with diverse COVID-19 vaccine adoption rates. Surprisingly, there was only minimal variability across states with respect to how people spent Thanksgiving suggesting that states with poorer vaccine coverage could witness a renewed surge in infections as social distancing norms are relaxed. In general, those who spent Thanksgiving with persons outside their household and/or traveled tended to be younger and were more often working outside the home in either essential or non-essential positions. Moreover, the age difference was even more striking among those who traveled for Thanksgiving and were highly active; the median age was 35 years in this group. That this group was relatively small may explain why the nationwide surge post-Thanksgiving was not even larger^5^. Our data demonstrate that had more persons traveled and participated in activities than this small group did, we would have anticipated twice as many cases. In contrast, if those who did travel behaved like the groups who did not, we would have expected a 50% reduction in cases.

It is important to note that there was a group of individuals who had Thanksgiving with people outside of their household or traveled but who did not report an increased prevalence of SARS-CoV-2 infection post-Thanksgiving. This group participated in few non-essential activities aside from their Thanksgiving gatherings. These data collectively suggest that it may be possible to mitigate the risks associated with travel and/or gatherings by limiting engagement in other activities. This is further supported by the modeled reductions in cases associated with limiting the number of activities and/or limiting visits to bars/restaurants and/or fitness activities. Specifically, the model suggests that there would have been a near 50% reduction in the number of primary cases if people could not visit bars and restaurants; a similar impact was shown for stopping visits to gyms. There has been much interest in understanding whether one venue confers more risk than another. We focused on modeling the impact of stopping visits to bars, restaurants, and gyms because in some ways these are venues where it is hardest to control the environment^8^.

Several limitations of this analysis must be acknowledged. Specifically, these data cannot be considered representative of the states; though we specified targets for recruitment that mirrored the demographic distributions of the states and applied analytic weights, online samples tend to underrepresent lower-income persons as well as those without access to the internet. However, we have previously demonstrated consistency with other types of samples with respect to outcomes like flu vaccination coverage^9,10^. Moreover, our primary purpose was to examine associations between behaviors and outcomes for which a representative sample is not required. All data is self-reported and subject to bias; however, we sampled participants in the weeks immediately following Thanksgiving and queried behaviors in the prior 2 weeks to minimize recall bias. All participants were not screened for SARS-CoV-2 and so, it is likely we missed undiagnosed infections; however, we observed similar associations with COVID-19 symptoms that were asked of all participants. We were also limited by not having detailed data on social contacts outside the household. As a result, we simplified this analysis and focused on only household-based contacts, which is unable to inform broader community-level transmission estimates. Our estimate of the number of cases in surveyed individuals was ~ 2× the number who tested positive despite fitting the probability of community transmission to the data. This discrepancy may be due to individuals testing positive following the survey date, pre-symptomatic and asymptomatic individuals^11^ who may also be less likely to be tested, or stochasticity inherent in the model. For example, of the 273 individuals who tested positive for SARS-CoV-2, the majority 85% (n = 230) reported symptoms.

Finally, we cannot conclusively say whether bars, restaurants, or gyms for example conferred more risk than other venues in part because individuals who were the most active tended to visit multiple types of venues. What these data do support is that restricting or even reducing the frequency of some non-essential activities rather than restricting all non-essential activities might help to minimize transmission. This is a particularly important message given that travel and gatherings are rapidly increasing with relaxation of social distancing and masking mandates. Unlike Thanksgiving and December where the majority were gathering at homes and with family, summer travel is likely to involve additional activities (e.g., visits to bars and restaurants while traveling). These findings suggest that it may be important to think beyond just travel itself to the activities that one participates in while traveling.

In conclusion, these data provide important information about how the combination of holiday travel and engagement in a high number of non-essential activities may fuel SARS-CoV-2 transmission. These data have important implications globally where vaccination rates remain well below 5% and even in settings where vaccines for COVID-19 are widely available such as the US. For example, in the US, vaccine coverage remains at only 50% and remains highly variable by state such that cases are once again surging. As viral variants which threaten vaccine efficacy continue to emerge^12,13^, there will be ongoing need to minimize infections in order to reduce the emergence of these variants^14^ particularly given unclear efficacy of vaccines against viral variants^15–17^. In this setting, other prevention strategies like minimizing large gatherings and high risk activities will remain critically important. We recommend that the focus of public health authorities should be on promoting messaging on how to travel and get together in a “low-risk” manner. Messages should incorporate language on how to mitigate risk by limiting the number and types of activities, in essence adopting an approach of harm reduction to prevent further surges.

## Methods

### Study sample

Participants were sampled across 10 US states: California, Florida, Illinois, Maryland, Massachusetts, Nebraska, North Dakota, South Dakota, Texas, and Wisconsin. States were chosen to represent diversity with respect to geography, policies/restrictions around mask use and social distancing as well as in case counts and SARS-CoV-2 test positivity. At the time of the survey, SARS-CoV-2 test positivity in these states ranged from 5.6% in Maryland to 41.5% in South Dakota (Supplementary Table 1).

Participants were recruited using Dynata (https://www.dynata.com), one of the largest first-party global data platforms. Dynata maintains a database of potential participants who are randomized to specific surveys if they meet the demographic targets of the survey; additionally, participants can select a survey from a list of potential options (survey topic not provided). Participants receive modest compensation for participation. Security checks and quality verifications include digital fingerprinting and spot-checking via third-party verification. In order to accrue demographically representative samples, we provided quotas for age, gender, race/ethnicity, and income based on the population composition of the states^9,10^. Individuals were surveyed from December 4–December 18, 2020, representing a range of 8–22 days after Thanksgiving. Participants provided informed consent, were 18 years of age or older, and a resident of the state they were sampled from. Sample sizes were proportional to the population size of the state with a range of 234 in North/South Dakota to 1490 in Texas for a total of 7905 across the 10 states. Across all states, 11,087 were routed to the survey; 493 did not start the survey, 625 started but did not complete the survey, and 588 responses were excluded for non-eligibility.

### Survey

The online survey was developed by the study team as part of a larger effort called the Pandemic Pulse study^9^. Specifically, the goal of this study was to assess changes over time in factors associated with SARS-CoV-2 transmission including mobility and adoption of non-pharmaceutical interventions such as masking and social distancing as well as changes with respect to SARS-CoV-2 testing access and uptake (all versions of the surveys conducted as part of this study are available from: github.com/sclipman/PandemicPulse). The survey version used for this wave of data collection captured information on demographics, people’s experiences with COVID-19 symptoms, and self-reported SARS-CoV-2 PCR testing in the past 2 weeks, and SARS-CoV-2 positivity among other household members in the prior 2 weeks. In terms of the holidays, people were asked about whether they had Thanksgiving outside of their home (traveled) and/or with individuals from outside their household and the size of their Thanksgiving gathering as well as their travel plans over the December holidays. Additionally, participants were asked about the types and frequency of recent activities (within the past 2 weeks). These were distinguished as essential (grocery store and/or pharmacy) and non-essential (bars both indoor and outdoor, restaurants both indoor and outdoor, gyms, participation in group-outdoor fitness classes, church, visits with friends and family, and salons either for a haircut or some other service). Finally, participants were asked about social distancing and mask use while participating in these activities as well as about general participation in gatherings of 10 or more and 100 or more persons.

### Statistical analysis

Characteristics of persons were compared by whether they had Thanksgiving at home with household members only, had Thanksgiving at home but with non-household members/guests, or had Thanksgiving away from their home/traveled. Categorical variables were compared using chi-squared tests and continuous variables were compared using a Kruskal–Wallis test. Survey weights were applied using iterative proportional fitting to further reflect the composition of the study states with respect to age, gender, race/ethnicity, income, and education using the Census Bureau's American Community Survey^18^. Weighting variables were raked according to their marginal distributions, as well as by two-way cross-classifications for each pair of demographic variables.

A total of 7770 persons answered the question about how they spent Thanksgiving and thus were included in the analysis. Univariable and multivariable logistic regression was used to identify factors associated with SARS-CoV-2 positivity (among those who received a test) and factors associated with COVID-19 symptoms in the weeks following Thanksgiving. We calculated an activity score which was a count outcome that summed the number of times an individual participated in non-essential activities in the prior 2 weeks, this included visits to bars, restaurants, places of worship, salons, theatres and stadiums, gyms, participation in group outdoor fitness activities and gatherings with friends and family. This activity score was further dichotomized into low (< 1 non-essential activity per day) and high (≥ 1 non-essential activity/day). Factors included in the multivariable model included factors associated with SARS-COV-2 positivity and COVID-19 symptoms and included age, gender, race/ethnicity, employment status, household size, and attending a gathering of 10 or more persons. The final multivariable model also adjusted for state of residence and included an interaction between where individuals had Thanksgiving and their activity score (low vs. high). Analyses were conducted using Stata 15.0 (College Station, Texas), R (v3.5.1) and Python (v3.8.5).

### Transmission model

Using data from all participants who were tested in the prior 2 weeks, we fit the probability of community transmission (${p}_{c}$) considering the activity score (truncated to a maximum of 35 activities) for each individual:

$${P}_{i}=1-{\left(1-{p}_{c}\right)}^{{A}_{i}}$$

Using the observed self-reported cases of SARS-CoV-2 and number of activities, we fit the value for p_c_ using Markov Chain Monte Carlo (MCMC) methods and an uninformed prior. In order to add estimates of uncertainty in our model, we ran multiple chains to provide a range of values. Specifically, we ran 100 simulations each with a chain length of 10,000 steps. This produced a range of estimates for p_c_ (mean = 0.033, min = 0.029, max = 0.036)^19^. Using the range of fit values, we then used the activity scores to estimate if each individual in the survey data was infected or not sampling from a binomial distribution with ${P}_{i}$ for each individual and an adjustment to account for the skew in the distribution of ${P}_{i}$ values (Supplementary Figure 2). To simplify the number of assumptions, we only considered the possibility of secondary cases within the individual’s household using self-reported data on household structure including the ages of all household members and a range of age-specific secondary attack rates^20^. Deaths were estimated using a range of age-specific infection fatality rates based on published data^21^. To simulate the impact of different behavioral patterns, we further considered several scenarios that altered the number and types of activities that individuals participated in. Specifically, we considered the following: (a) all participated in the same distribution (resampled) of activities as those who had Thanksgiving at home with household members only; (b) all participated in the same distribution of activities as those who traveled for Thanksgiving; (c) all could only participate in up to 7 activities regardless of type; (d) all could only participate in up to 14 activities regardless of type; (e) all could participate in all activities except for gyms/outdoor fitness; and (f) all could participate in all activities except for bars/restaurants (indoors and outdoors).

### Ethical clearance

The study was approved by the Institutional Review Board of the Johns Hopkins Bloomberg School of Public Health (IRB00012413) and was conducted in accordance with all relevant ethical guidelines and regulations. All participants consented to participate.

## Supplementary Information

Supplementary Information 1.

Supplementary Information 2.
