# Supplementary material for: Holiday gatherings, mobility and SARS-CoV-2 transmission: results from 10 US states following Thanksgiving
Source: Sci Rep. 2021 Aug 30;11:17328. doi: 10.1038/s41598-021-96779-6 (PMC8405672; doi:10.1038/s41598-021-96779-6)
Supplement: Supplementary file 1 — Supplementary Information 1. [file 41598_2021_96779_MOESM1_ESM.docx]

**Supplementary Table 1. SARS-CoV-2 test positivity in sampled states for the week of December 15, 2020.**

|  | SARS CoV-2 test positivity | Range (+/-) |
| --- | --- | --- |
| State of Residence | ·· | ·· |
| Massachusetts | 5·6 | 1·7 |
| Maryland | 6·3 | 2·4 |
| Illinois | 8·4 | 4·7 |
| Wisconsin | 11·7 | 12·6 |
| Florida | 9·7 | 9·7 |
| Texas | 17·6 | 17·6 |
| California | 13·1 | 13·1 |
| Nebraska | 9·6 | 7·0 |
| North Dakota | 6·6 | 4·4 |
| South Dakota | 41·5 | 12·1 |

**Supplemental Table 2. Factors associated with self-reported SARS-CoV-2 test positivity and self-reported symptoms in the prior 2 weeks. Results from univariable logistic regression analysis.**

|  | OR for SARS-CoV-2 positivity (95% CI)† | OR for COVID-19 symptoms (95% CI) |
| --- | --- | --- |
| Age (per 5 years) | 0·90 (0·86, 0·95) | 0·84 (0·82, 0·86) |
| Male gender | 1·57 (1·18, 2·09) | 1·09 (0·94, 1·27) |
| Race/Ethnicity | ·· | ·· |
| White | 1 | 1 |
| Black/African American | 0·95 (0·61, 1·48) | 1·37 (1·09, 1·73) |
| Hispanic/Latino | 1·32 (0·97, 1·81) | 2·04 (1·72, 2·41) |
| Asian/Pacific Islander | 1·01 (0·49, 2·10) | 0·65 (0·43, 0·97) |
| Other | 0·64 (0·18, 2·28) | 1·51 (0·87, 2·61) |
| Household size (per person) | 1·11 (1·02, 1·20) | 1·09 (1·05, 1·13) |
| Employment | ·· | ·· |
| Employed, working at home | 1 | 1 |
| Not employed | 0·81 (0·47, 1·41) | 0·81 (0·65, 1·02) |
| Working outside the home (non-essential) | 1·36 (0·86, 2·14) | 1·25 (1·0, 1·57) |
| Working outside the home (clinical care) | 2·80 (1·78, 4·43) | 4·25 (3·30, 5·46) |
| Working outside the home (other essential) | 2·79 (1·78, 4·37) | 2·78 (2·19, 3·53) |
| Attending a gathering of 10 or more persons* | 1·60 (1·47, 1·74) | 1·57 (1·50, 1·65) |
| Interaction between Thanksgiving & activity score** | ·· | ·· |
| Household members only, low activity | 1 | 1 |
| Non-household members only, low activity | 1·25 (0·73, 2·11) | 1·17 (0·93, 1·46) |
| Outside home, low activity | 0·98 (0·57, 1·66) | 1·23 (0·98, 1·55) |
| Household members only, high activity | 1·27 (0·35, 4·61) | 2·63 (1·50, 4·63) |
| Non-household members, high activity | 4·03 (2·07, 7·87) | 4·45 (3·11, 6·35) |
| Outside home, high activity | 12·1 (7·86, 18·7) | 15·6 (12·3, 19·6) |
| State of residence | ·· | ·· |
| Massachusetts | 1 | 1 |
| Maryland | 1·68 (0·81, 3·46) | 1·51 (1·0, 2·26) |
| Illinois | 3·11 (1·58, 6·13) | 1·89 (1·31, 2·73) |
| Wisconsin | 1·75 (0·81, 3·77) | 1·78 (1·20, 2·64) |
| Florida | 3·02 (1·57, 5·81) | 2·19 (1·52, 3·16) |
| Texas | 3·69 (2·03, 6·73) | 2·35 (1·66, 3·31) |
| California | 2·58 (1·41, 4·74) | 2·33 (1·65, 3·28) |
| Nebraska | 2·27 (0·92, 5·57) | 2·30 (1·44, 3·68) |
| North and South Dakota | 0·61 (0·13, 2·88) | 1·72 (0·99, 2·98) |
| OR, unadjusted odds ratio; CI, confidence interval  †Among 962 tested in prior 2 weeks  *Reflects behavior in the prior 2 weeks and excludes Thanksgiving dinner  **Activity score was calculated as the sum of all non-essential activities in the prior 2 weeks. Low activity was considered a frequency less than once/day and high activity was a frequency of once a day or more | | |

**Supplementary Figure 1.** Sankey diagram showing COVID-19 symptoms, SARS-CoV-2 PCR testing, and self-reported positivity in the prior 2 weeks by Thanksgiving behavior. Participant responses are depicted in each rectangular node with flows proportional to how many individuals report that pattern of behavior. Green flows represent respondents who did not travel or have Thanksgiving with anyone outside their immediate household; orange flows represent those who had Thanksgiving in their home with at ≥ 1 person from outside their household; red flows represent those who had Thanksgiving outside their home.

**Supplementary Figure 2.** The distribution of $P_{i}$ values with the mean $p_{c}$ value and reported activity values.

**Supplementary Data 1:** Modeled estimates of the number of cases in participants, household members, and deaths of household members from 100 stochastic simulations based on various levels of activities.
